# Presentation and Management of Pyogenic Liver Abscess in a 23-Week Pregnant Woman

**DOI:** 10.1155/2013/845215

**Published:** 2013-10-01

**Authors:** Beril Yüksel, Ali Seven, Suna Kucur, Ilay Gözükara, Nadi Keskin

**Affiliations:** Department of Obstetrics and Gynecology, Faculty of Medicine, Dumlupinar University, 43100 Kutahya, Turkey

## Abstract

Pyogenic liver abscess during pregnancy is an extremely rare condition. We report a case of 33-year-old, 23-week pregnant woman with pyogenic liver abscess. She was still in the hospital for medical observation of fever, when a sudden episode of tachycardia with a pulse of 210 beats per minute and tachypnea with a respiratory rate of 30 breaths per minute was encountered. At that moment, her fever was 39.6°C (103.28 Fahrenheit). The abdominal ultrasound stated a calcific echogenic mass with a measure of 6 cm in the liver region. Given the sonographic characteristics noted, a liver abscess was suspected. Our case was successfully treated with an ultrasound guided percutaneous aspiration of the abscess and a wide spectrum antibiotic. At 38 weeks of gestation, an elective cesarean delivery was performed. The female neonate weighed 3200 g with APGAR scores of 9 and 9 at the first and fifth minutes, respectively.

## 1. Introduction

Pyogenic liver abscess (PLA) complicating pregnancy is extremely rare. The most common microorganisms reported with this complication are *Escherichia coli* and *Bacteroides* spp. and polymicrobial infections [1]. Adequate management of this unusual clinical situation requires early diagnosis and treatment.

In this report, we present an interesting case of a pregnant woman who had complaint of fever and developed septic shock because of PLA, an unusual focus of infection.

## 2. Case Report

A 33-year-old patient, gravida 1, presented to the emergency service with fatigue and complaint of fever at home. From her medical history, we learned that she was hospitalized for gastroenteritis and fever for 10 days in another hospital, and before her admission to our clinic, she was discharged the day before with oral second generation cephalosporin treatment. The diagnosis in her previous hospitalization was gastroenteritis and nephrolithiasis. The patient stated that she had intermittent fever at home after her discharge and was not feeling well.

In her admission, she denied any dysuria, diarrhea, sore throat, coughing, nausea, or rupture of membranes. In ultrasound, she had a healthy fetus of 23 weeks of gestation. She said she had fever of 39.8°C at home, but in her admission; it was 37°C (98.6 Fahrenheit) with a blood pressure of 110/70 mm Hg, a pulse of 80 beats per minute, and a respiratory rate of 18 breaths per minute. Her lungs were clear except for shallow respiratory sounds on the right. Her abdomen was nontender. The cervical examination was also normal with no discharge or discomfort. The initial laboratory tests were all normal including blood count, urine, and stool analysis. The only abnormal test was elevated C-reactive protein level (19.96 mg/dL).

 A few hours later, while she was still in the hospital for medical observation, a sudden episode of tachycardia with a pulse of 210 beats per minute and tachypnea with a respiratory rate of 30 breaths per minute was encountered. At that moment, her fever was 39.6°C (103.28 Fahrenheit). Her electrocardiography was interpreted as sinus tachycardia. Her echocardiography revealed mild tricuspid regurgitation, first degree of pulmonary hypertension, and normal systolic functions. Her chest X-ray was normal. Cultures from blood, urine, stool, and cervix were taken and she was monitorized in the intensive care unit (ICU). Meanwhile, meropenem (Meropenem, 1 gram/day, intravenously) was started.

**Figure 1:**
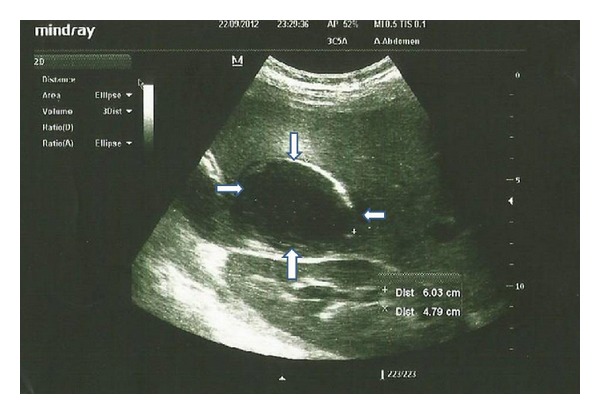
The abdominal ultrasound image of calcified wall of the abscess in 23-week pregnant woman.

 In ICU, her blood pressure was 70/40 mm Hg and her pulse was 140 beats per minute. An hour later, she had a recent onset of shortness of breath and a chest pain. Her d-dimer was above 100.000. Her white blood count was 25000, and her platelet value was 77000. Her partial oxygen pressure was 77; partial carbon monoxide pressure was 20 with a pH value of 7.47. Low molecular weight heparin was started for a possible pulmonary thromboembolism or disseminated intravascular coagulation. An ultrasound was ordered for suspicion of deep venous thrombosis. The ultrasound report was normal for venous system but incidentally stated a calcific echogenic mass with a measure of 6 cm in the liver region (Figure 1). Given the sonographic characteristics noted, a liver abscess was suspected.

 Platelet suspensions were ordered and the patient was consulted with a general surgeon. An ultrasound guided percutaneous aspiration and drainage with sterile saline was done when her platelet count was 55.000. The next day, her blood pressure and pulse rate were high normal and her fever was stabilized after 48 hours. The culture from blood and stool was positive for *E. coli.* All laboratory tests returned to normal within a week. She was discharged from hospital after 7 days of meropenem therapy. 

 She continued her routine pregnancy visits after her discharge. At 38 weeks of gestation, an elective cesarean delivery was performed. The female neonate weighed 3200 g with APGAR scores of 9 and 9 at the first and fifth minutes, respectively. 

## 3. Discussion

Pyogenic liver abscess is a serious, life threatening condition that is difficult to diagnose and treat. Clinical suspicion is important because of its high mortality rate [1]. Although fever and right upper quadrant abdominal pain are known to be the most common symptoms, the clinical presentation in many cases is nonspecific and is difficult to diagnose. The most common microorganisms reported with this clinical entity are *Escherichia coli* and *Bacteroides* spp. [1]. Usually, a mucosal defect within the digestive tract is blamed for bacteria invasion into the portal system followed by hematogenous spread to the liver [2].

Just like the clinical symptoms, the laboratory tests are also non-specific for diagnosis of PLA. The most frequent findings are increased ALP, leukocytosis, and increased fibrinogen which are not specific during pregnancy. Elevated ALT and infection-induced thrombocytopenia are also reported [3]. The sensitivity of ultrasound for the diagnosis of PLA is reported to be 85.8% [4]. The clinical incidence of PLA varies from region to region but has been reported to be 11 cases per million persons per year [5]. 

PLA during pregnancy is an extremely rare condition which represents a diagnostic and therapeutic challenge. As the clinical and laboratory findings are usually nonspecific, a misdiagnosis is often possible, but an early diagnosis and therapy are vital because of its high perinatal mortality rate in untreated cases. Another problem with PLA onset in pregnancy is the possible progression of the disease to severe sepsis or septic shock which is associated with increased rates of preterm delivery, fetal infection, multiple organ dysfunction syndrome, and death. 

Sepsis is the situation of systemic inflammatory response syndrome (SIRS) because of an infection. SIRS is defined as the presence of two or more of the following: temperature greater than 38°C or less than 36°C, pulse greater than 90 beats/min, respiratory rate greater than 20 breaths/min, partial carbon monoxide pressure less than 32 mm Hg, and white blood cell count greater than 12,000/mm^3^ or less than 4,000/mm^3^ [6]. Sometimes, SIRS and sepsis may progress to multiple organ dysfunction and septic shock, which is a more serious and mortal complication. 

As the sepsis and septic shock during pregnancy can be fatal, an immediate empiric initiation of a large spectrum antibiotic treatment is vital. But even with the appropriate antibiotic selection and adequate fluid resuscitation, the prognosis is poor, unless the target of the infection is found. Especially in cases of abscess formation in certain tissues or organs, the treatment can be useless if the source of the infection is not drained or excised. 

 Differential diagnosis of sepsis is important. It tends to occur from specific sources, such as respiratory infections, that are the most common causes of sepsis, genitourinary, and abdominal sources of infection with primary bacteremia and unknown sources being the next most common causes [7]. In pregnancy, premature rupture of membranes and chorioamnionitis also should be considered. In our case, the patient suffered from gastroenteritis before her admission to our hospital. After deterioration of her clinic in the hospital, SIRS and septic shock with hypotension were settled. At first, all our attempts of finding a septic focus failed. But when we examined the right upper quadrant, we could identify a pyogenic abscess in the liver. Our case was successfully treated with an early diagnosis and prompt treatment. After her discharge, her routine visits revealed normal results and she gave birth to a healthy full-term baby.

## 4. Conclusion

Although rare, in situations of sepsis or septic shock in pregnancy, as well as the common sources of infection, a possibility of a liver abscess should also be kept in mind and ultrasonographic evaluation of this region should be considered.
